# Evaluation of the performance of health extension workers on malaria rapid diagnostic tests and predictor factors in Bahir Dar Zuria district, northwest Ethiopia: A cross-sectional study

**DOI:** 10.1371/journal.pone.0249708

**Published:** 2021-04-08

**Authors:** Zelalem Dejazmach, Getaneh Alemu, Mulat Yimer, Chalachew Muluneh, Banchamlak Tegegne

**Affiliations:** 1 Department of Medical Laboratory Science, College of Health Sciences, Woldia University, Woldia, Ethiopia; 2 Department of Medical Laboratory Science, College of Medicine and Health Sciences, Bahir Dar University, Bahir Dar, Ethiopia; 3 Medical Parasitology, Amhara Public Health Institute, Bahir Dar, Ethiopia; 4 Medical Parasitology and Vector Control, Bahir Dar Zuria *Woreda* Health Office, Bahir Dar, Ethiopia; Arizona State University, UNITED STATES

## Abstract

**Background:**

In Ethiopia, anti-malaria treatment is initiated after parasitological confirmation using blood film microscopy at health centers and hospitals, or serological rapid diagnostic tests at health posts. At health posts, the diagnosis is performed by health extension workers using rapid diagnostic tests after little training. However, there is paucity of data about the health extension workers’ performance on rapid diagnostic tests. Hence, periodic monitoring of the performances of health extension workers on malaria rapid diagnostic tests and predicted factors plays a pivotal role for the control of malaria.

**Methods:**

A cross sectional study was conducted in May 2020, among 75 health extension workers working at health posts in Bahir Dar Zuria district, Northwest Ethiopia. Their performance on malaria rapid diagnostic tests was assessed by distributing known positive and negative samples as confirmed by investigators using both rapid diagnostic test and blood film microscopy. Test results from health extension workers were then compared with that of investigators. Procedural errors committed while performing the tests were assessed using observational checklist. Data were analyzed using SPSS software version 20.

**Results:**

The overall sensitivity and specificity of health extension workers in detecting *Plasmodium* species were 96.8% and 98.7%, respectively with 97.3% result agreement between the health extension workers and investigators (kappa value = 0.949). The most common procedural errors committed by health extension workers was ‘not checking expiry date of the test kits’ followed by ‘not adhering to the appropriate time of reading results’ that 70.7% and 64% of the participants committed these errors, respectively. Total number of procedural errors committed by those who have got in-service training was decreased by 47.3% as compared to those without in-service training.

**Conclusions:**

Health extension workers had high performance on malaria rapid diagnostic tests. However, in-service training and periodic supervision should be given in order to maximize performance on these tests.

## Background

Malaria is a vector borne disease caused by protozoan parasites of the genus *Plasmodium* [1]. Five species namely *Plasmodium falciparum* (*P*. *falciparum*), *Plasmodium vivax* (*P*. *vivax*), *Plasmodium malarae* (*P*. *malarae*), *Plasmodium ovale* (*P*. *ovale*), and *Plasmodium knowlesi* (*P*. *knowlesi*) naturally infect humans [2]. Of these, *P*. *falciparum* and *P*. *vivax* are the most prevalent species globally [3].

According to World Health Organization (WHO) 2019 report, malaria is one of the most common diseases with major global public health problem. About 228 million malaria cases occurred in 2018 causing 405, 000 deaths worldwide. Most malaria cases and deaths occur in African Region, which accounted for 93% (213 million) and 94% (380,000) of all malaria cases and deaths, respectively in 2018 [3].

In Ethiopia, about 68% of the population is at risk of the disease and there are 1–2 million annual confirmed malaria cases. There were 356 reported deaths in 2017 [1]. *P*. *falciparum* and *P*. *vivax* are the two dominant species accounted for 69% and 30% of infections, respectively [4]. Malaria transmission in Ethiopia primarily occurs at altitudes below 2,000 meters above sea level and is mainly seasonal with the major and minor transmission season from September to December and April to May, respectively [5].

The WHO recommends that anti-malarial treatment should be initiated after parasitological confirmation of suspected cases [6]. As a result, early laboratory diagnosis using blood film microscopy is indispensable in order to reduce transmission, morbidity, and mortality due to malaria. This can be done in health institutions equipped with clinical laboratory; otherwise, rapid diagnostic tests (RDTs) are important alternatives. Although microscopic diagnosis of malaria remains the gold standard, currently the use of RDTs has gained major importance wherever microscopy is not available [7]. Despite, the presence of various malaria RDTs that are commercially available, all detect *Plasmodium* species antigens. Hence, they are based on the detection of either histidine-rich protein 2 (HRP2), a protein synthesized by *P*. *falciparum* only and lactate dehydrogenase (LDH) or aldolase, enzymes produced by all human *Plasmodium* species [8, 9].

Rapid diagnostic tests have a significant impact on the reduction of malaria burden in the world [10]. Since they are relatively simple to perform and interpret the results, rapid in release of results, require limited training, and allow for the diagnosis of malaria at the community level [8, 11] without the need of electricity with reasonable sensitivity and specificity [8]. As a result, RDTs are being used by health extension workers (HEWs) at health posts in Ethiopia. In order to increase the accessibility of health care services to the community, the Health Extension Program (HEP) in Ethiopia was initiated in 2003 with the main objectives of prevention and control of communicable diseases like malaria [12].

The HEP is implemented at health post or community level which is the lowest level of the Ethiopian health tier system. The HEWs are community health workers who are trained for one year in order to provide basic health services such as family planning, vaccination, health education, diagnosis and treatment of certain diseases like diarrhea and malaria. In this program, two female HEWs are assigned to a health post in each *Kebele* (village). Health extension workers are recruited from the communities in which they will work according to specific criteria: they are female (except in pastoralist areas), at least 18 years old, have completed at least grade 10 in education, and speak the local language. Females are selected because most of the HEP packages are related to issues affecting mothers and children; since communication is thought to be easier between mothers and female HEWs [13].

The main objective of the HEP in Ethiopia is to improve access to essential health services provided at the village and household levels [13]. Despite the main task of HEWs is to work on disease prevention, they are also involved in diagnosis and treatment of common diseases like malaria. As a result, at health posts where there is no laboratory and laboratory professionals, HEWs are given little training and screen febrile cases with RDTs before prescribing anti-malaria drugs. They treat patients with uncomplicated malaria using artemisinin combination therapy, chloroquine, and primaquine based on the national malaria diagnosis and treatment guideline [14].

Despite malaria RDTs are easy to perform with little training, strictly following the manufacturer’s instructions is important to have reliable results. Hence, periodic monitoring of HEWs while performing the RDT procedures is mandatory to take timely corrections, in case errors are committed. However, there is paucity of data about the HEWs’ performance on malaria RDTs in Ethiopia. Therefore, the present study aimed to assess the performances of HEWs on malaria RDTs and predicting factors in Bahir Dar Zuria district, where malaria is a public health problem.

## Methods and materials

### Study design, area and period

A cross-sectional study was conducted at all health posts in Bahir Dar Zuria district, Northwest Ethiopia in May 2020. Bahir Dar Zuria district is one of the 14 districts in West Gojjam Zone, which is located at a distance of 560 km from capital city of the country (Addis Ababa) and it is situated surrounding Bahir Dar city, capital city of Amhara National Regional State. The altitude of the district ranges from 1700–2300 meters above sea level [15]. The district has an average annual rainfall of 1035 mm. The minimum and maximum temperature lies at 10°C and 32°C, respectively [15]. Likewise, the major transmission season of malaria occurs from September to December followed by April to May with minor transmission [14]. The district has 36 *kebeles* and 9 health centers and 36 health posts where 81 HEWs have been employed.

#### Study participants’ recruitment

All HEWs who were actively on duty during data collection period and gave consent to participate in the study were included.

### Data collection

Data on predicting factors for committing procedural errors while performing RDTs were collected using structured questionnaire. The questionnaire was designed to collect information about HEWs’ age (in years), prior experiences in performing malaria RDTs (in years), prior in-service refreshment training on malaria RDTs (yes/no), supply of RDTs to the health post so that the service has not been interrupted (yes/no), frequency of supervisions per quarter year by the malaria officer or other responsible body assigned to support HEWs in the diagnosis and treatment of malaria (number of visits per every 3 months), trust in RDT result by HEWs or their feelings in the accuracy of RDT results (trust/not trust).

The HEWs’ performances on RDTs were evaluated using known positive and negative samples as confirmed by investigators using both RDT and blood film microscopy. Two ml of venous blood was collected by the principal investigator from laboratory confirmed malaria cases for *P*. *falciparum (pf)*, *P*. *vivax (PV)* and *P*. *falciparum/P*. *vivax* (pf/PV) mixed infections and from apparently healthy (non-malaria infected) individuals. Each of the collected blood samples was transferred into Ethylenediamine Tetra Acetic Acid containing test tubes. Seven blood samples (two *P*. *falciparum*, two *P*. *vivax*, one mixed, and two negative) confirmed by both microscopy and RDT were distributed to 34 health posts and HEWs tested all samples using their own RDTs. Then data on the performance of each procedural step were collected using observational checklist prepared for this purpose. The prepared checklists divided the RDT procedure into 8 steps in three domains: procedural (checking RDT expired date), safety (discarding the pipette in the sharps box), as well as accuracy (labeling of RDT, dispensing correct volume of blood, dispensing blood in correct well, dispensing correct volume of buffer, time of result reading, and correctly reporting the result). Among the seven samples to be run by each HEW, one RDT procedure (4^th^ sample) was strictly observed by principal investigator, noting whether the HEWs performed each step correctly or not.

### Data analysis

Data were checked for completeness and entered and analyzed in statistical package for social science (SPSS) software version 20. Descriptive statistics like frequency and percentage were manipulated to explain the study participants and their test performance. Multivariable Poisson regression was fit to test for associations between error count data and pre-specified covariates (age group, prior RDT experiences in years, prior in-service training on RDT, regular supply of RDTs to the health post, frequency of supervision per quarter year, trust in RDT result). Selected pre-specified covariates were included in the analysis based on the hypothesized relationship to the outcome. P-value < 0.05 in the multivariate analysis was considered as statistically significant at 95% confidence level. Kappa value was also calculated to show the strength of test agreement between ‘HEWs and investigators’ and interpreted as follow: values ≤ 0 as indicating no agreement and 0.01–0.20 as none to slight, 0.21–0.40 as fair, 0.41–0.60 as moderate, 0.61–0.80 as substantial, and 0.81–1.00 as almost perfect agreement [16].

Performance of HEWs’ on malaria detection and species identification using RDTs were tested by computing the sensitivity, specificity, positive predictive values (PPV) and negative predictive values (NPV) against investigators’ results using Med-Calc software version 19.3. The software calculates the value for each parameter with the sense of the following equations.

sensitivity = a/n1×100

specificity = b/n2×100

positive predictive value = a/n3×100

negative predictive value = b/n4×100

where n1 = number of true positives identified by investigator;

n2 = number of true negatives confirmed by investigator;

n3 = number of samples identified as positives by HEWs;

n4 = number of samples identified as negatives by the HEWs;

a = number of cases identified as positives by investigator and by the HEWs;

b = number of samples identified as negatives by investigator and by the HEWs.

### Ethical consideration

The research was carried out after ethical approval was obtained from the institutional review committee of College of Medicine and Health Sciences, Bahir Dar University with reference number CMHSc009/2020. Additionally, supportive letters were obtained from Amhara public health institute, West Gojjam Zone health department, and Bahir Dar Zuria district health office and permission was obtained from each health center authorities where clinical samples used to evaluate HEWs were collected. Information obtained at any course of the study was kept confidential. Informed verbal consent was obtained from participating HEWs and no personal and health facility identifier was included as participants were given a unique code. According to the research ethics guideline of Bahir Dar University, informed verbal consent is accepted to conduct researches where the data collection doesn’t include invasive procedures (like aspirate or biopsy samples). Hence, the verbal consent for the present study was approved by the institutional review committee. Health extension workers who committed procedural errors while performing RDTs were given feedback on the spot about the procedural errors they committed and how those errors could be avoided.

## Results

### Socio-demographic characteristics of study participants

Among the total of 81 HEWs employed in Bahir Dar zuria district, 75 participated in the study. The remaining 6 HEWs were not actively on work during data collection period due to different reasons. In this study, age of the participants ranged from 25–34 with mean age of 29.23 (± 2.077 SD) years. Forty-five (60.0%) participants have taken in-service training on malaria RDTs. The participants had work experience ranged from 5–14 years with mean experience of 8.96 (±2.5 SD) years (Table 1).

**Table 1:** Socio-demographic characteristics and malaria RDT related experiences of HEWs working at health posts in Bahir Dar Zuria district, Northwest Ethiopia in May 2020 (N = 75).

Characteristics	Categories	Frequency (%)
Age group in years	25–29	42 (56.0)
	30–34	33 (44.0)
Prior experience in performing RDT (in years)	5–9	41 (54.7)
	10–14	34 (45.3)
Prior in-service training on malaria RDT	Yes	45 (60.0)
	No	30 (40.0)
Regular supply of RDTs to the health post	Yes	42 (56.0)
	No	33 (44.0)
Frequency of supervision per quarter year	1	20 (6.7)
	2	11 (14.7)
	3	44 (58.7)
Trust in malaria RDT result	Yes	55 (73.3)
	No	20 (26.7)

### Performance of health extension workers

From a total of 75 HEWs participated in the study, all the 8 procedural steps was carried out correctly by more than 70% of the participants, except for steps 1,5 and 7. The median number of total and accuracy steps completed correctly was 6 and 4, respectively. The number of errors observed ranged from 0 to 5 errors per HEW. The steps most commonly performed incorrectly were step 1 and 7 (Table 2).

**Table 2:** Procedural performance of HEWs in performing RDTs in Bahir Dar Zuria district, Northwest Ethiopia in May 2020 (N = 75).

Number	Tasks	Categories	Frequency	Percent
1	Check RDT expired date	Yes	22	29.3
		No	53	70.7
2	Labeling of RDT	Yes	60	80.0
		No	15	20.0
3	Dispense correct volume of blood	Yes	69	92.0
		No	6	8.0
4	Dispense blood in correct well	Yes	75	100.0
		No	0	0.0
5	Discards the pipette in the sharps box	Yes	51	68.0
		No	24	32.0
6	Dispense correct volume of buffer	Yes	63	84.0
		No	12	16.0
7	Keeps exact time of result reading	Yes	27	36.0
		No	48	64.0
8	Correctly report the result	Yes	68	90.7
		No	7	9.3
Summary statistics (range)		**Mean (SD)**	**Median (IQR)**	
**Total steps correctly completed (0–8)**		5.81(1.249)	6(6)	
**Accuracy steps correctly completed (0–6)**		4.32(0.961)	4(4–5)	

### Interpretation of RDT results

A total of 525 (375 positive and 150 negative) samples were tested by HEWs. Of these, 511(97.3%) were correctly reported. Among 375 positive samples, 363 (96.8%) were correctly reported as positive and the rest 9(2.4%) were falsely reported as negative. Out of 75 samples with mixed infection, 72(96.0%) were correctly reported as mixed and the remaining 3 (4.0%) were falsely reported as *P*. *falciparum*. From the 150 negative samples, 148(98.7%) were correctly reported as negative (Table 3).

**Table 3:** Performance of HEWs in interpreting RDT results in Bahir Dar Zuria district, Northwest Ethiopia in May 2020 (N = each of 75 HEWs interpreting test results for 7 samples).

**Test**		Investigators’ RDT results				
		*P*. *falciparum*	*P*. *vivax*	Mixed infection	Negative	Total
**HEWs’ RDT results**	*P*. *falciparum*	147		3	1	151(28.8%)
	*P*. *vivax*		144		1	145(27.6%)
	Mixed infection			72		72(13.7%)
	Negative	3	6		148	157(29.9%)
	Total	150(28.6%)	150(28.6%)	75(14.3%)	150(28.6%)	525(100%)

The overall sensitivity and specificity of HEWs ‘on detection of *Plasmodium* species were 96.8% and 98.7%, respectively while the overall positive and negative predictive values were 99.5% and 94.3%, respectively. The test agreement between the HEWs and investigators was 97.3% with kappa value of 0.949 (Table 4).

**Table 4:** Sensitivity, specificity, predictive values, and agreement between HEWs’ and investigators’ RDT results on detection and identification of *Plasmodium* species at health posts in Bahir Dar Zuria district, Northwest Ethiopia in May 2020 (N = 75).

Parasite	Sensitivity (%)	Specificity (%)	PPV (%)	NPV (%)	Kappa value
*P*. *falciparum*	98.0	99.7	99.3	99.2	0.981
*P*. *vivax*	96.0	99.7	99.3	98.4	0.967
Mixed infections	96.0	100.0	100.0	99.3	0.976
Overall *Plasmodium* species	96.8	98.7	99.5	94.3	0.949

### Poisson regression of factors associated with total error count

According to Poisson regression model, the outcome of total error count showed that in-service training on RDT was associated with the overall test performance. The mean error ratio (MER) for getting in-service training was 0.527(95% CI 0.365–0.763), which corresponds to the mean number of total errors for those who took in- service training being 47.3% (95% CI 23.7–63.5) lower when compared to those without training (Table 5).

**Table 5:** Covariates associated with total error counts using multivariable Poisson regression among HEWs working at health posts in Bahir Dar Zuria district, Northwest Ethiopia in May 2020 (N = 75).

Characteristics	Categories	Frequency (%)	MER(95% CI)	P-value
Age group in years	25–29	42 (56.0)	0.897(0.588–1.369)	0.614
	30–34	33 (44.0)	1	
Prior experience in performing RDT (in years)	5–9	41(54.7)	0.905(0.590–1.387)	0.645
	10–14	34(45.3)	1	
Prior in-service training on malaria RDT	Yes	45(60.0)	0.527(0.365–0.763)	0.001
	No	30(40.0)	1	
Regular supply of RDTs to the health post	Yes	42(56.0)	0.933(0.656–1.328)	0.700
	No	33(44.0)	1	
Trust in malaria RDT result	Yes	55(73.3)	1.044(0.719–1.516)	0.821
	No	20(26.7)	1	
Frequency of supervision (number per quarter year)	1	20(26.7)	1.037(0.693–1.552)	0.859
	2	11(14.7)	1.349(0.837–2.173)	0.219
	3	44(58.7)	1	

## Discussion

Malaria rapid diagnostic tests have been important alternatives to diagnose malaria at health posts in Ethiopia as the tests can be performed by HEWs with little training. However, monitoring of the RDTs performance of HEWs in conducting the tests is indispensable to maximize the diagnostic quality of RDTs. In the present study, the performance of HEWs on RDTs were evaluated and reported for the first time in Ethiopia as far as our knowledge is concerned.

Health extension workers performed and interpreted RDT test results almost perfectly with sensitivity and specificity of 96.8% and 98.7%, respectively on detection and identification of *Plasmodium* species. The sensitivity of the present study was higher than the study reported in Western Kenya (92%). Such higher sensitivity in the present study might be due to variations in duration of experience in performing RDTs. However, the specificity of the present finding was comparable with previous result of 97.0% in Western Kenya [17].

As compared to the investigators’ result, there were two negative samples reported as false positive (one *P*. *falciparum* and one *P*.*vivax*), nine positive samples as false negative, and three samples with mixed infection as *P*. *falciparum* mono infection. These false positive and false negative results might be due to shipment and storage conditions of the RDT kits such as direct exposure to sunlight. Study conducted in Burkina Faso documented that transportation and storage condition of RDTs affect the test performance [18]. Moreover, false positive results might be due to delayed result reading. In the current study there were twelve observations where result readings were made after 20 minutes. In support of this, a study conducted in Damot Gale district, Southern Ethiopia found that HEWs believe that reactive results are visible after 20 minutes and they wait longer time to report reactive results [19].

The false negative results might be due to early result reading and dispensing inadequate volume of blood in addition to inappropriate storage conditions. Reporting of mixed infections as *P*. *falciparum* mono infections might be due to difficulty in the interpretation of faint positive test lines. Previous studies have also reported difficulties in the interpretation of faint-positive test lines by HEWs [20, 21]. In our observation, there was also interpretation of two invalid results as negative (data not shown). A study from Zambia also reported that community health workers read faint positive and invalid results of RDTs as negative suggesting that subjective interpretation may contribute to false results [22]. These phenomena might be caused by excess amount of blood, prolonged exposure to hot temperature as well as transportation conditions of the RDT kits [23].

The accuracy of RDTs can be affected by incorrect blood volumes, incorrect buffer volume and timing of the test interpretation. Excessive blood volumes can result in staining and obscuring test lines [24] and results in prozone effect [9]. This could cause a health worker to misread a positive result as negative, especially with a low parasite density infection where test lines tend to be faint. Inadequate blood volumes can also reduce test sensitivity by producing false negative results when antigen quantity is insufficient to generate a visible test line [24]. Excessive buffer volumes can also result false-positive results due to non-specific bindings. While, insufficient volume of buffer can lead invalid test result by impeding clearance of the strip and/or slow down migration with failure to generate a control line [25].

During our observation, we have noted that only two and four HEWs added too little and too much blood, and one and eleven HEWs added too little and too much buffer, respectively. This has a significant impact when viewed from the number of patients each HEW is serving every day.

Early and delayed reading of RDTs result was another common error observed in the present study as thirty-six and twelve HEWs have committed those errors, respectively. Previous studies have also noted that reading RDT results early is a frequently observed error [17, 21, 26]. Reading too early may cause false-negative results, while reading too late may cause false-positive results due to a backflow phenomenon [25, 27].

In-service training on RDTs was found to be significantly associated with lower procedural error rates. Getting training decreased the mean number of total procedural errors by 47.3% (95% CI 23.7–63.5) as compared to those without training. This finding is supported by previous studies showing that training on RDTs is associated with improved performance [17, 18]. Moreover, another study underlines the need to provide at least minimal training prior to RDTs use, even for community health workers with prior healthcare experience [20].

## Limitations of the study

Performance of HEWs in patient preparation and sample collection were not evaluated. The optimal condition of RDT such as storage conditions was not observed. Moreover, the presence of observers may have impacted HEWs’ performance that formal observation can lead to a state of anxiety that decrease or increases in performance during supervision and evaluation [28, 29].

## Conclusions

Health extension workers in the present study area had good performance on malaria RDTs. However, the errors seen during observations are significant when viewed from the number of patients screened by each HEW every day. The most common procedural errors committed by HEWs were ‘not checking expiry date of the test kits’ and ‘not adhering to the appropriate time of reading results’. Prior training on malaria RDTs significantly affects the performance of HEWs in correctly completing each step of the test procedure. Regular supervision and in-service training should be given for HEWs on RDT procedures by cluster health centers in order to reduce errors observed in the present study. Further research should be conducted to investigate performance of HEWs’ in patient preparation and sample collection.

## Supporting information

S1 File (ZIP)

## Abbreviations

FMOH: Federal Ministry of Health
HEP: Health Extension Program
HEWs: Health Extension Workers
NPV: Negative Predictive Value
PPV: Positive Predictive Value
RDTs: Rapid Diagnostic Tests
WHO: World Health Organization
