# Supplementary material for: Evaluation of the performance of health extension workers on malaria rapid diagnostic tests and predictor factors in Bahir Dar Zuria district, northwest Ethiopia: A cross-sectional study
Source: PLoS One. 2021 Apr 8;16(4):e0249708. doi: 10.1371/journal.pone.0249708 (PMC8031431; doi:10.1371/journal.pone.0249708)
Supplement: S1 File — (ZIP) [file pone.0249708.s001.zip › Zipped files/Questionnaire and checklist.docx]

| **Questionnaire and checklists for health extension workers’ RDT performance evaluation** | | | | |
| --- | --- | --- | --- | --- |
| Identification code ---------------------------------- | | | | |
| **No.** | **Questionnaire** | **Responses** | | |
| 101 | Age (in years) |  | | |
| 102 | Educational background |  | | |
| 103 | Have you ever used mRDT to diagnose malaria? | 1. Yes 2. No | | |
| 104 | Duration of Practice with RDTs (in Years) |  | | |
| 105 | Is there regular supply of RDTs without interruption? | 1. Yes 2. No | | |
| 106 | Do you trust mRDT result? | 1. Yes 2. No | | |
| 107 | Do you trust in malaria RDT result? | 1. Yes 2. No | | |
| 108 | Have you ever been trained for malaria RDT? | 1. Yes 2. No | | |
| 109 | Frequency of supervision you get per quarter year |  | | |
| **Check list** | | Response | |  |
|  |  | Yes | No |  |
| 110 | Availability of guidelines/standard operating procedure |  |  | |
| 111 | Read RDT expiration date |  |  | |
|  | Label RDTs |  |  | |
| 112 | Dispense correct volume of blood |  |  | |
| 113 | Dispense blood in correct well |  |  | |
| 114 | Discards the pipette in the sharps box |  |  | |
| 115 | Dispense correct volume of buffer |  |  | |
| 116 | Keeps exact time of result reading |  |  | |
| 117 | Delayed result reporting |  |  | |
| 118 | Early result reporting |  |  | |
| 119 | Correctly report the results |  |  | |
